# Small but mighty: Functional landscape of the versatile geminivirus-encoded C4 protein

**DOI:** 10.1371/journal.ppat.1009915

**Published:** 2021-10-07

**Authors:** Laura Medina-Puche, Anelise F. Orílio, F. Murilo Zerbini, Rosa Lozano-Durán

**Affiliations:** 1 Department of Plant Biochemistry, Centre for Plant Molecular Biology (ZMBP), Eberhard Karls University, Tübingen, Germany; 2 Dep. de Fitopatologia/BIOAGRO, Universidade Federal de Viçosa, Viçosa, Brazil; 3 National Research Institute for Plant-Pest Interactions, Universidade Federal de Viçosa, Viçosa, Brazil; 4 Shanghai Center for Plant Stress Biology, CAS Center for Excellence in Molecular Plant Sciences, Chinese Academy of Sciences, Shanghai, China; Virginia Polytechnic Institute and State University, UNITED STATES

## Abstract

The fast-paced evolution of viruses enables them to quickly adapt to the organisms they infect by constantly exploring the potential functional landscape of the proteins encoded in their genomes. Geminiviruses, DNA viruses infecting plants and causing devastating crop diseases worldwide, produce a limited number of multifunctional proteins that mediate the manipulation of the cellular environment to the virus’ advantage. Among the proteins produced by the members of this family, C4, the smallest one described to date, is emerging as a powerful viral effector with unexpected versatility. C4 is the only geminiviral protein consistently subjected to positive selection and displays a number of dynamic subcellular localizations, interacting partners, and functions, which can vary between viral species. In this review, we aim to summarize our current knowledge on this remarkable viral protein, encompassing the different aspects of its multilayered diversity, and discuss what it can teach us about geminivirus evolution, invasion requirements, and virulence strategies.

## Introduction

Viruses are acellular intracellular parasites that invade and subvert host cells in order to multiply their nucleic acid genomes and spread. With the aim to create a cellular environment permissive for viral replication, viruses manipulate and usurp the cellular molecular machinery through the action of proteins they encode, which interact with, modify, redirect, inhibit, interfere with, utilize, or hijack host proteins. Owing to the fast replication and elevated mutation rate of viruses, viral proteins evolve quickly and are constantly exploring their potential functional landscape; this, in turn, enables the high-paced adaptation of viruses to their hosts.

**Fig 1:**
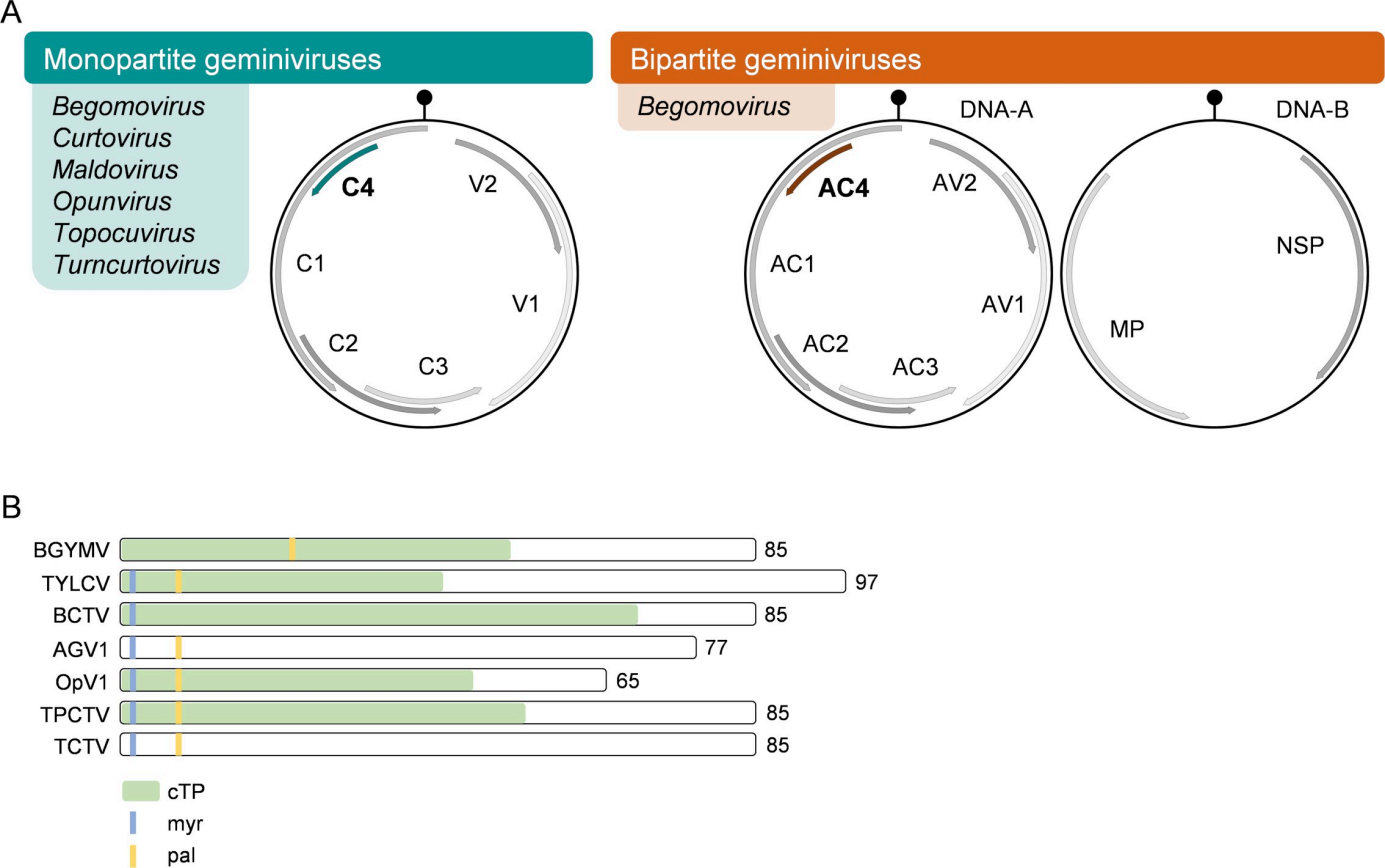
General features of the C4/AC4 protein encoded by geminiviruses. **(A)** Genome organization of geminiviruses. **(B)** Features of selected C4/AC4 proteins. Protein size in aa is indicated on the right. The cTP was predicted by ChloroP (http://www.cbs.dtu.dk/services/ChloroP/) [72]. The N-terminal myr site and the pal site were predicted by GPS-Lipid (http://lipid.biocuckoo.org/webserver.php) [73]. For further information, see S1 and S2 Tables. cTP, predicted chloroplast transit peptide; myr, predicted myristoylation site; pal, predicted palmitoylation site.

Geminiviruses are insect-transmitted plant viruses with circular single-stranded (ss) DNA genomes, causal agents of devastating crop diseases around the globe. This viral family (Geminiviridae) is divided in 14 genera, based on genome structure, host range, and insect vector: *Becurtovirus*, *Begomovirus*, *Capulavirus*, *Citodlavirus*, *Curtovirus*, *Eragrovirus*, *Grablovirus*, *Maldovirus*, *Mastrevirus*, *Mulcrilevirus*, *Opunvirus*, *Topilevirus*, *Topocuvirus*, and *Turncurtovirus* ([1]; https://talk.ictvonline.org/ictv-reports/ictv_online_report/ssdna-viruses/w/geminiviridae). Most geminiviruses described to date are members of the genus *Begomovirus* (currently, 445 out of 520), which encompasses both monopartite and bipartite viruses (with genomes composed of 1 or 2 circular ssDNA molecules, respectively); viruses in all other genera are exclusively monopartite. All geminiviral genomic components described to date are approximately 2.5 to 3.2 kb in size, and complete genomes are described to encode between 4 and 8 proteins through bidirectional and partially overlapping open reading frames (ORFs) located in both virion and complementary strands (Fig 1A). Geminiviruses can associate with satellite molecules, which sometimes provide additional proteins acting as virulence factors (reviewed in [2]).

The invasion of the host plant cell by a geminivirus requires a series of essential steps that are not yet fully understood; these include intra- and intercellular movement, suppression of antiviral defenses, DNA replication, viral gene expression, and acquisition and transmission by the insect vector. To ensure a successful infection, certain essential functions need to be provided by viral proteins; these can be facilitated by different proteins in different viruses and/or accomplished through distinct virulence strategies, but they cannot be lost during evolution. Since the geminiviral proteome is limited, the viral proteins are most likely multifunctional, as has been demonstrated in numerous instances.

Monopartite begomoviruses encode 6 proteins, namely Rep/C1, TrAP/C2, REn/C3, C4, V2, and CP/V1 (Fig 1A). In bipartite begomoviruses, positional homologues of these proteins (named Rep/AC1, TrAP/AC2, REn/AC3, AC4, AV2, and CP/AV1) are found in one of the genomic components, DNA-A, while the other component, DNA-B, encodes a nuclear shuttle protein (NSP) and a movement protein (MP). Functional information of geminivirus-encoded proteins is rather fragmentary; nevertheless, the function of the viral proteins encoded by certain positional homologues seems to be frequently conserved across geminiviruses in different genera. That is the case for the replication-associated protein (Rep/C1/AC1), which reprograms the cell cycle and enables replication of the viral DNA, or of REn/C3, which is described to act as a replication enhancer (reviewed in [3,4]). Along the same lines, V2 acts as a silencing suppressor in all geminiviruses tested to date (reviewed in [5]), and CP forms the viral capsid and acts as the NSP in monopartite geminiviruses (reviewed in [3]). TrAP/C2, on the other hand, seems to have broader functional diversity among geminiviruses (including transcriptional activation, suppression of posttranscriptional gene silencing (PTGS)/transcriptional gene silencing (TGS), or manipulation of hormone signaling, among other activities), despite displaying a conserved nuclear or nuclear/cytoplasmic localization (reviewed in [5]). An unparalleled case of functional diversity, nevertheless, is provided by the geminiviral C4/AC4 protein. C4 is the smallest geminiviral protein described to date (approximately 10 KDa), has been proven essential for full infectivity in all geminiviruses tested, and is described as a symptom determinant (e.g., [6–17]). C4 is encoded in the complementary strand of the viral genome, and, in begomoviruses, its ORF is completely embedded in that of the viral Rep (Fig 1A). Strikingly, and despite the obvious evolutionary constraints that this overlap entails, C4 is the most diverse protein in this viral family (Fig 1B), and, in stark contrast to all other geminivirus-encoded proteins, is consistently subjected to positive selection instead of purifying selection [18–22]. Interestingly, acquisition of alternative C4 sequences by recombination has been proven to give rise to new and more virulent geminiviruses [16,23] and correlates with epidemiologically relevant properties like ability to break resistance [24,25] or independence from a satellite molecule [16].

C4 displays a broad diversity of functions during the viral infection, both within and between geminiviruses, which is perhaps just beginning to emerge. In this review, we intend to summarize our current understanding of the C4 protein encoded by geminiviruses, considering its main features, its evolutionary properties, and the functions ascribed to it to date, and to discuss what it can teach us about the geminivirus infection cycle and its interaction with the host plant.

## Main features of the geminivirus-encoded C4 protein

Members of the genera *Begomovirus*, *Curtovirus*, *Maldovirus*, *Opunvirus*, *Topocuvirus*, and *Turncurtovirus* are described to encode a C4 protein (referred to AC4 in bipartite begomoviruses; previously also known as L4/AL4) (Table 1). The C3 protein encoded by *Capulavirus*, *Grablovirus*, and *Topilevirus* members is a C4 homologue; in members of other genera, namely *Becurtovirus*, *Mastrevirus*, and *Mulcrilevirus*, C4 positional homologues with similar features to those previously described can also be predicted (Table 1), suggesting that the prevalence of C4 among geminiviruses might be higher than initially thought.

**Table 1:** Features of the C4 proteins encoded by the type species of the different Geminivirus genera.

Genus	Species	Isolate	Accession number	RefSeq number	Virus abbreviation	Gene name / protein ID	cTP	myr	pal	kDa	aa
*Becurtovirus*	*Beet curly top Iran virus*	Iran/Kerman/2005/A	EU273818	NC_010417	BCTIV	NA / NA		G2	C4	14,9	134
*Begomovirus*	*Bean golden yellow mosaic virus*	Dominican Republic/1987	DNA-A: L01635; DNA-B: L01636	DNA-A: NC_038791; DNA-B: NC_038790	BGYMV	NA / NA	52		C23	9,8	85
	*Tomato yellow leaf curl virus* (*)	Spain/Almeria/Pepper/1999	AJ489258	NC_004005	TYLCV	C4 / NP_658996.1	43	G2	C8	11,2	97
*Capulavirus*	*Euphorbia caput-medusae latent virus*	South Africa/Dar10/2010	HF921459	-	EcmLV	C3 / CCV02661.1				13,6	121
*Citlodavirus*	*Passion fruit chlorotic mottle virus* (*)	Brazil/CDS_MS_BR/2014	MG696802	NC_040706	PCMoV	-					
*Curtovirus*	*Beet curly top virus*	United States/CA/Logan /1985/California Logan	M24597	NC_001412	BCTV	C4	69	G2		9,7	85
*Eragrovirus*	*Eragrostis curvula streak virus*	South Africa/Gre1/g261/2007/A	FJ665631	NC_012664	ECSV	-					
*Grablovirus*	*Grapevine red blotch virus*	456]17NOV2010/2010	JQ901105	-	GRBV	NA / NA	8		C9, C46	17,9	160
*Maldovirus*	*Apple geminivirus 1*	-	KM386645	-	AGV1	C4 / AJZ68901.1		G2	C8	8,7	77
*Mastrevirus*	*Maize streak virus*	South Africa/A	Y00514	NC_001346	MSV	NA / NA	3	G7	C21	5,3	47
*Mulcrilevirus*	*Mulberry crinkle leaf virus*	JS/2015	KR131749	-	MCLV	NA / NA		G8	C12	12,9	113
*Opunvirus*	*Opuntia virus 1* (*)	DBG_14_1	MN100000	-	OpV1	C4 / QHU79453.1	47	G2	C8	7,3	65
*Topilevirus*	*Tomato apical leaf curl virus* (*)	AR:Yuto:Tom419:08/2017	MG491195	-	ToALCV	C3 / AUF71984.1	23	G9	C20	18,1	159
*Topocuvirus*	*Tomato pseudo-curly top virus*	US/Florida/1994	X84735	NC_003825	TPCTV	C4 / NP_620736.1	54	G2	C8	9,5	85
*Turncurtovirus*	*Turnip curly top virus*	Iran/ Zafarabad/B11/2006/A	GU456685	NC_014324	TCTV	C4 / YP_003778179.1		G2	C8	9,7	85
Unclassified	*Polygala garcinii geminivirus 1*	South Africa/1-1/2012	MG001959	NC_037068	PgGV1	NA / NA	44	G2		10,7	97

Asteriks indicate non-type species. Protein size in kDa and in aa is indicated. cTP, predicted chloroplast transit peptide; myr, predicted myristoylation site; pal, predicted palmitoylation site. The cTP was predicted by ChloroP (http://www.cbs.dtu.dk/services/ChloroP/) [72]. The N-terminal myr site and the pal site were predicted by GPS-Lipid (http://lipid.biocuckoo.org/webserver.php) [73]. For scores and cutoffs, please see S1 Table.

The geminiviral C4 proteins described so far, all encoded in the complementary strand, fully or partially overlapping with the Rep/C1 ORF but in a different frame, range from approximately 7 to approximately 11 KDa in size and share an identity that can be as low as <20%, being the least conserved geminiviral protein (see examples in Fig 1B; Table 1). The diversity of the geminiviral C4 proteins is reflected in the varied, not fully overlapping subcellular localization of the proteins encoded by different geminiviruses, as well as in the multiple functions and differential interactors (Table 2) that have been assigned to the positional homologues in different geminiviruses.

**Table 2:** Described plant interactors of C4/AC4 proteins.

Protein	Species	Virus abbreviation	Known interactor(s)	Known function(s)	Reference(s)
C4	*Tomato Leaf Curl Guandong virus*	ToLCGdV	BAM1	Suppression of intercellular spread of silencing	[9]
C4	*Beet severe curly top virus*	BSCTV	CLV1, PEPR2	N/A	[8,54]
C4	*Tomato yellow leaf curl virus*	TYLCV	BRI1, PSKR1, CLV1, BRL3, BAM1, BAM2, BAM3, FLS2, NIK1, CAS	Suppression of intercellular spread of silencing; suppression of SA-mediated defenses; weak PTGS supression in bean	[26,41,49,50,66]
C4	*Tomato Leaf Curl Yunnan virus*	TLCYnV	HIR1, NbSKη, BKI1, NbDRM2	Suppression of HIR1-dependent cell death; reactivation of the cell cycle; impairing MAPK activation; TGS suppression	[10,48,60,61]
AC4	*Cotton leaf curl Multan virus*	CLCuMV	SAMS	Suppression of TGS and PTGS	[57]
C4	*Tomato leaf curl virus*	ToLCV	SlSK	N/A	[33]
C4	*Beet curly top virus*	BCTV	AtSKη	Activation of cell cycle	[30,31]
AC4	*Mungbean yellow mosaic virus*	MYMV	BAM1	Suppression of intercellular spread of silencing	[28]

HIR1, HYPERSENSITIVE INDUCED REACTION 1; MAPK, mitogen-activated protein kinase; PTGS, posttranscriptional gene silencing; SA, salicylic acid; TGS, transcriptional gene silencing.

**Fig 2:**
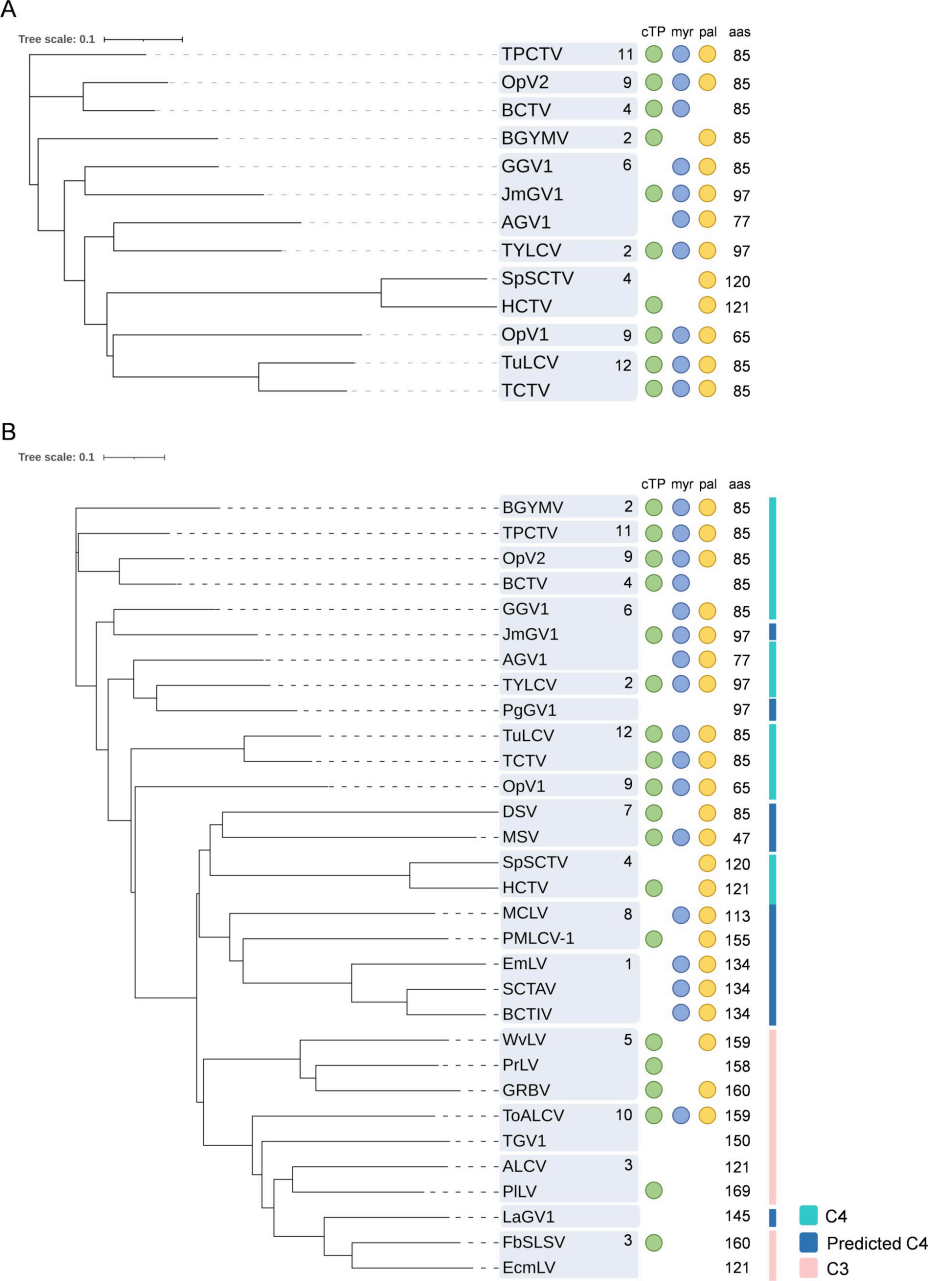
Phylogenetic tree of C4/AC4 proteins and presence/absence of predicted cTP and myr/pal sites. Annotated C4/AC4 proteins were used in **(A)**; in **(B)**, potential proteins encoded by predicted ORFs with positional and sequence homology to C4 (predicted C4; dark blue) as well as *Capulavirus*, *Grablovirus*, and *Topilevirus* C3 proteins (pink) are included. In light blue, annotated C4/AC4 proteins. The presence or absence of predicted cTP, myr, and pal is indicated; protein size in aa is shown on the right. Numbers within the gray boxes correspond to different Geminivirus genera as follows: 1, *Becurtovirus*; 2, *Begomovirus* (BGYMV: NW; TYLCV: OW); 3, *Capulavirus*; 4, *Curtovirus*; 5, *Grablovirus*; 6, *Maldovirus*; 7, *Mastrevirus*; 9, *Opunvirus*; 10, *Topilevirus*; 11, *Topocuvirus*; and 12, *Turncurtovirus*. The gray boxes without name correspond to unclassified viruses. For further information, see S1 and S2 Tables. cTP, predicted chloroplast transit peptide; myr, predicted myristoylation site; ORF, open reading frame; OW, Old World; NW, New World; pal, predicted palmitoylation site.

Several motifs potentially determining protein localization can be identified in C4 proteins, namely a myristoylation (myr) site, a palmitoylation (pal) site, and a chloroplast transit peptide (cTP) (see examples in Fig 1B). N-myristoylation is an irreversible protein lipidation, consisting in the covalent attachment of a myristoyl group, derived from myristic acid, to an N-terminal glycine residue. Pal, on the other hand, is a reversible protein lipidation that frequently occurs on cysteine residues. Myr and pal, which often coexist in the same protein, increase hydrophobicity, hence favoring membrane association. The cTP acts as a chloroplast targeting sequence and can be highly divergent in length, composition, and organization; this amino acid sequence is cleaved upon protein import to the organelle through the translocon (TOC/TIC) complexes, releasing the mature protein to the interior of the chloroplast. Lipidations have been proven for the C4 protein from tomato yellow leaf curl virus (TYLCV) and C4 from beet severe curly top virus (BSCTV) in vivo [8,26] and for that of tomato leaf curl Yunnan virus (TLCYnV) in vitro [27], while the relevance of the potential myr and pal sites for protein localization and/or function has been shown in several other cases [26,28–30]. Intriguingly, these localization signals seem to have been either lost or gained multiple times during evolution, and all possible combinations can be found in nature (Fig 2).

As a consequence of the diversity in the combinatorial presence of targeting signals, as well as, possibly, interacting partners, geminiviral C4 proteins can display an array of subcellular localizations, including plasma membrane, chloroplasts, nucleus, and cytoplasm (e.g., [9,26–29]); these subcellular localizations can be dynamically regulated at different stages of the infection [26,27]. The observed differences in subcellular localization will necessarily impact the interactome and functionality of individual C4 proteins, as illustrated in the following sections.

In addition to lipidations, C4 is subjected to another posttranslational modification, namely phosphorylation [27,30,31]; this modification might rely, at least partly and/or in some cases, on the interaction with the plant kinase SKη/BIN2, a well-known signaling component originally described as a negative regulator of the brassinosteroid (BR) signaling pathway [32], which seems to be common among C4 proteins [27,30,31,33,34]. Phosphorylation has proven essential for the developmental effects of C4 from BCTV [30]; in combination with myr, phosphorylation controls the nuclear/cytoplasmic shuttling of C4 from TLCYnV, hence its activity [27] (see below).

## Evolutionary aspects of C4/AC4 proteins

The phylogeny of C4/AC4 sequences is incongruent with the whole-genome phylogeny. While the whole-genome phylogeny of geminivirids shows a clear separation of members of the different genera [1] and divides begomoviruses into 2 strongly supported clades including viruses from the Old World (OW) and New World (NW) [35], the clusters observed in C4/AC4-based phylogenies include both OW and NW begomoviruses as well as members of different Geminiviridae genera (Fig 2; see also Fig 3 in [18] and Fig 2 in [36]). This incongruence could be due to the location of *C4*/*AC4* in a recombination hot spot [37]. Thus, the evolutionary history of C4/AC4 proteins may be distinct from those of the other geminiviral proteins. As mentioned above, acquisition of alternative C4 sequences by recombination provides a selective advantage [16,23–25], which is consistent with the multiple roles assigned to C4/AC4 proteins in the geminiviral infection cycle.

One interesting aspect of C4/AC4 proteins is that they are the only geminiviral protein to be consistently under positive selection. This was initially noted for the OW monopartite begomovirus cotton leaf curl Multan virus (CLCuMV; [20]) and later shown to be the true also for other OW and NW monopartite begomoviruses such as TYLCV [22] and tomato leaf deformation virus (ToLDeV; [18]). A recent study analyzed data sets of 200 randomly chosen begomoviruses (one sequence from each virus) and of 11 curtovirus isolates, and found that, in both cases, C4/AC4 was under positive selection, while the overlapping portion of C1/AC1 was under purifying selection [36]. We have analyzed species data sets of 21 mono- and bipartite begomoviruses, and C4/AC4 is under positive selection in 13 of them (Table 3). Four additional species data sets had values of approximately 0.9, indicating close to neutral selection. Moreover, no other gene is under positive selection in 20 of the 21 species data sets, the single exception being AV2 of mungbean yellow mosaic India virus (MYMIV) (Table 3). We also calculated the dN/dS values for each of the 3 domains (cTP, myr, and pal), and they are generally similar to the values for the full C4 sequence (S3 Table). Positive selection was observed in viruses infecting cultivated (bean golden mosaic virus, BGMV; TYLCV) or noncultivated hosts (euphorbia yellow mosaic virus, EuYMV; macroptilium yellow spot virus, MaYSV), with narrow (African cassava mosaic virus, ACMV; CLCuMV) or wide (MaYSV) host ranges, and was not dependent on the number of sequences in the data set. Thus, while positive selection may not be a universal feature of C4/AC4 genes, they are indeed under positive selection in many geminiviruses, unlike all the other geminiviral genes. This is even more remarkable since, as mentioned above, the *C4*/*AC4* gene is located in a recognized recombination hot spot [37]. Inasmuch as recombination contributes to the genetic variability of geminiviruses, it also plays an important role in purging deleterious mutations, which are mostly, if not entirely, nonsynonymous. Evidently, such purging effect is not acting on C4/AC4. A clear example MaYSV, in which AC4 is under strong positive selection (dN/dS = 1.4500) even though it is located in a recombinant fragment [38]. This recombinant fragment is responsible for the high nucleotide diversity of the MaYSV DNA-A, but this diversity is expressed mostly as synonymous mutations in Rep (dN/dS = 0.2248) and nonsynonymous mutations in AC4 [38].

**Table 3:** Nonsynonymous to synonymous substitution ratios (dN/dS) calculated for OW and NW begomoviruses.

Virus	Acronym	Group^a^	Number of sequences	AC4	Rep	TrAP	Ren	CP	AV2
						
African cassava mosaic virus	ACMV	OW	252	**2.0018**	0.1479	0.4342	0.4342	0.0975	0.2263
Ageratum enation virus	AgEV	OW	44	0.8989	0.2337	0.6903	0.2753	0.0627	0.1705
Bhendi yellow vein India virus	BhYVIV	OW	52	**1.1987**	0.2827	0.4495	0.5048	0.1453	0.2363
Bhendi yellow vein mosaic virus	BhYVMV	OW	75	**1.4870**	0.2125	0.4355	0.4392	0.1277	0.2233
Cotton leaf curl Gezira virus	CLCuGV	OW	81	**1.4433**	0.1576	0.5735	0.2560	0.1010	0.1705
Cotton leaf curl Multan virus	CLCuMV	OW	129	**1.4449**	0.2351	0.8385	0.3717	0.1419	0.3584
East African cassava mosaic virus	EACMV	OW	207	**1.4322**	0.1280	0.3743	0.6368	0.1432	0.2463
Mungbean yellow mosaic India virus	MYMIV	OW	85	0.5750	0.1345	0.3776	0.5309	0.0872	**1.1188**
Squash leaf curl China virus	SLCCNV	OW	38	**1.4377**	0.1171	0.7540	0.2530	0.0582	0.3112
Sweet potato leaf curl virus	SPLCV	OW	142	**1.3603**	0.2221	0.6123	0.3561	0.0868	0.1281
Tomato leaf curl New Delhi virus	ToLCNDV	OW	547	0.7223	0.1339	0.6691	0.2742	0.0594	0.3297
Tomato leaf curl Taiwan virus	ToLCTV	OW	54	0.9286	0.2107	0.4516	0.2385	0.0870	0.1519
Tomato yellow leaf curl China virus	ToYLCCNV	OW	39	0.9372	0.1566	0.4063	0.3554	0.0387	0.1818
Tomato yellow leaf curl Thailand virus	TYLCTV	OW	42	0.7963	0.2115	0.2937	0.4280	0.0387	0.2175
Tomato yellow leaf curl virus	TYLCV	OW	763	**2.0298**	0.2540	0.5549	0.4206	0.1204	0.2327
Bean golden mosaic virus^b^	BGMV	NW	117	**2.5300**	0.2490	0.4007	0.4261	0.1037	-^c^
Blainvillea yellow spot virus^b^	BlYSV	NW	30	0.8575	0.1837	0.4378	0.1902	0.0282	-
Euphorbia yellow mosaic virus^b^	EuYMV	NW	50	**1.1500**	0.2589	0.6470	0.2710	0.0611	-
Macroptilium yellow spot virus^b^	MaYSV	NW	21	**1.4500**	0.2284	0.5240	0.3078	0.0943	-
Tomato leaf deformation virus	ToLDeV	NW	72	0.5515	0.2699	0.9475	0.2292	0.0748	-
Tomato severe rugose virus	ToSRV	NW	74	**1.8367**	0.1133	0.3310	0.2407	0.1646	-

^a^OW, Old World; NW, New World.

^b^From Xavier and colleagues [21]

^c^NW begomoviruses do not have an AV2 gene.

Values >1, which are indicative of positive selection, are highlighted in bold.

It has been posited that overlapping genes may limit the accumulation of synonymous substitutions or a higher accumulation of nonsynonymous substitutions in one of the overlapping genes, leading to an artifactual increase in the dN/dS ratio [39]. However, C2/AC2 and C3/AC3 also overlap, and both genes are under purifying selection (Table 3). Therefore, the >1 dN/dS values for C4/AC4 can be correctly interpreted as positive selection acting upon this gene.

Although the evolutionary and functional significance of positive selection in C4/AC4 genes of geminiviruses is unknown, it is reasonable to assume that it is related to the multiple, accessory roles of C4/AC4 in the geminiviral infection cycle, as well as their large number of interacting partners. The fact that several of the roles played by C4/AC4 can also be performed by other geminiviral proteins may release it from evolutionary constraints, allowing it greater freedom to explore the amino acid sequence space. In particular, positive selection has been implicated in the counterresponse of viruses against host defense responses based on RNA silencing [40], a role that has been assigned to C4/AC4 proteins. Nevertheless, it is remarkable that such a small protein can tolerate a relatively large number of nonsynonymous mutations. In this regard, it is noteworthy that C4/AC4 proteins were found to be intrinsically disordered proteins [36], which are characterized precisely for tolerating nonsynonymous mutations and for interacting with multiple cellular partners.

## The broad functional landscape of C4

### Functions of C4 at the plasma membrane

Multiple geminiviral C4/AC4 proteins have been described as associated to the plasma membrane, a localization that requires their lipidation [12,26–29,41]. At the plasma membrane, at least some C4/AC4 proteins can concentrate in plasmodesmata, the microscopic channels providing cytoplasmic and membrane continuity between plant cells [28,41]; the presence of C4/AC4 at the cell periphery has prompted long-standing speculations regarding its involvement in viral movement, a role that would be consistent with its plasmodesmal localization. Nevertheless, the experimental evidence supporting a movement role of C4 during the geminiviral infection is scarce and occasionally based on the observation that a null mutant is impaired in systemic invasion despite retaining its ability to replicate [15,42], a feature later shown applicable to other viral proteins. Arguing against an essential role in viral cell-to-cell movement, C4 from TYLCV has limited capacity to move and/or to mediate macromolecular trafficking intercellularly, at least in mesophyll and epidermal cells of *Nicotiana benthamiana* or *Arabidopsis* [12,41], and null mutations in C4 do not always result in impaired systemic infections [43–47]. At the plasma membrane, C4/AC4 from different geminiviruses has been shown to interact with receptor-like kinases (RLKs) or associated proteins and potentially interfere with their activity, possibly inhibiting signal transduction. The interaction of C4 from TLCYnV with the inhibitor BRI1 KINASE INHIBITOR 1 (BKI1) impairs its dissociation from the RLK ERECTA (ER), suppressing the autophosphorylation of the latter and the downstream activation of mitogen-activated protein kinases (MAPK) cascades and their function in antiviral defense [48]. The C4 proteins from TYLCV and tomato leaf curl Guangdong virus (ToLCGdV) and the AC4 protein from mungbean yellow mosaic virus (MYMV) interact with BARELY ANY MERISTEM 1 (BAM1) (and, at least for C4 from TYLCV, with its closest homologue BAM2); in the case of TYLCV and MYMV, this interaction seems to be particularly strong at plasmodesmata [9,28,41]. BAM1 has been proposed to promote the intercellular movement of silencing [41,49]; given that the silencing spread is inhibited by these C4/AC4 proteins, an activity for which their plasma membrane localization is an essential requirement, it has been speculated that the viral proteins might be suppressing this particular function of BAM1. Interestingly, other viral proteins have been recently shown to interact with BAM1, suggesting a central role of this RLK in plant–virus interactions [50,51].

A close homologue of BAM1, CLAVATA 1 (CLV1), interacts with C4 from BSCTV, which may interfere with the downstream signaling pathway [8]. This same C4 protein also interacts with Pep1 RECEPTOR 2 (PEPR2), one of 2 receptors of the plant endogenous peptide Pep1 [52–54]. Pep1 acts as a damage-associated molecular pattern (DAMP), and its perception activates DAMP-triggered immunity (reviewed in [55]). Strikingly, overexpression of PEPR2 and its activation by exogenously applied Pep1 promotes the internalization of C4 from the plasma membrane, and has an antiviral effect [54], which prompts the speculation that PEPR2 might modify C4 to trigger its plasma membrane release and therefore interfere with its virulence function. Of note, phosphorylation-dependent detachment of N-myristoylated proteins from the plasma membrane occurs in animals [56], raising the idea that PEPR2 might phosphorylate and free C4 following perception of Pep1 and activation of its intracellular kinase domain.

The physical association of C4/AC4 proteins with the intracellular domain of RLKs seems to be an extended phenomenon; whether these interactions are specific for a given C4/AC4-RLK combination, or, on the contrary, C4/AC4 proteins target the conserved kinase domain of RLKs and hence can broadly bind members of this protein family is at this point unclear. Nevertheless, the finding that C4 from BSCTV interacts with both CLV1 and PEPR2 [8,54] and that C4 from TYLCV can associate with a number of RLKs from *Arabidopsis* [50] seems to favor the latter; certain C4/AC4-RLK interactions might have been selected for in specific geminivirus/plant pathosystems.

### C4 as silencing suppressor

Some C4/AC4 proteins have been described to function as suppressors of gene silencing, both TGS and PTGS. As mentioned above, some C4/AC4 proteins specifically interfere with the cell to cell or systemic spread of silencing [5,9,28,41]. The C4/AC4 proteins from African cassava mosaic virus (ACMV), EACMV, CLCuMV, and tomato leaf curl virus-Australia (ToLCV) suppress PTGS [29,33,57,58]. At least for ACMV, this property might be associated to its ability to bind small RNA (sRNA) [59]. In the case of ToLCV, binding of the protein to the host shaggy-like kinase (SlSK) through its carboxyl terminus seems essential for its silencing suppressing capacity [33]. The activity of C4 from CLCuMuV depends on its interaction with the core enzyme in the methyl cycle S-adenosyl methionine synthetase (SAMS), which underlies its capability to act as both PTGS and TGS suppressor [57]. The C4 protein from TLCYnV also functions as a TGS suppressor, in this case by interacting with and impairing DNA binding of the DNA methyltransferase DOMAINS REARRANGED METHYLASE 2 (DRM2) [60].

### C4 manipulates defense responses

Another common theme in the function of geminivirus-encoded C4/AC4 proteins is the suppression of different aspects of plant defense, which is not restricted to antiviral gene silencing. As previously mentioned, at the plasma membrane, C4 from TLCYnV suppresses the activation of MAPK cascades, which restrict viral accumulation, through the interaction with BKI1 [48]; in addition, this protein abolishes the HYPERSENSITIVE INDUCED REACTION 1 (HIR1)-mediated cell death following a double approach: by impairing the HIR1 homotypic interaction, essential for the onset of cell death, and by inducing the accumulation of the negative regulator of HIR1 LRR1 [61]. C4/AC4 from TYLCV, BCTV, and EACMV can relocalize from the plasma membrane to the chloroplast upon perception of a biotic threat at the cell surface, and impair the downstream activation of chloroplast-dependent defenses, including salicylic acid (SA) accumulation and SA-dependent responses, which are proven to play an antiviral defense [26,62].

### C4 as symptom determinant

The geminiviral C4/AC4 protein has long been described as a symptom determinant (e.g., [11,14,17,63,64]). Transgenic expression of C4/AC4 in plants leads to dramatic developmental phenotypes, which can vary between viruses (e.g., [10,31,63,65,66]) but frequently include leaf curling, organ twisting, and darker green color in photosynthetic tissues. C4 is the major determinant of the distinctive vein swelling associated to BCTV infection [14,63,64], and its expression in transgenic plants results in ectopic cell division [30,31,63,65]. Constitutive expression of C4 from BSCTV in *Arabidopsis* induces the expression of cell cycle–related genes and leads to callus formation or ectopic cell divisions, suggesting that at least one of the activities underlying the capacity of C4 to prompt developmental alterations is the reactivation of the cell cycle [67,68]. The reactivation of the cell cycle by the C4 protein from BCSTV seems to occur through the induction of *RKP*, a ubiquitin E3 ligase [67]; recently, the symptom determinant ability of this C4 has been attributed to its capacity to bind CLV1 in the shoot apical meristem [8], although whether the virus will reach this area during a natural infection remains to be determined. However, it is possible to generate stable transgenic plants constitutively expressing C4 from other geminiviruses (e.g., TYLCV), which suggests that either not all of them induce cell dedifferentiation and division or their efficacy and/or underpinning molecular mechanisms differ.

In the case of TLCYnV, C4 promotes cell division by interacting with NbSKη (the homologue of *Arabidopsis* BIN2 in *N*. *benthamiana*) [10,27,34]; interestingly, the interaction between C4 and BIN2/NbSKη is prevalent in the geminivirus family [30,31,33,34,69]. C4 from TLCYnV shuttles NbSKη from its native nuclear localization to the cell periphery, in turn triggering the accumulation of the NbSKη substrate Cyclin D1;1, an effect that stimulates reentry in cell cycle and ultimately cell division [10,27].

C4/AC4 proteins have been shown to interfere with hormone synthesis and/or signaling, specifically those of BR, auxin, and SA [26,50,69,70], an effect that may underlie or contribute to the impact of these viral proteins on development. Supporting this notion, application of exogenous BR alleviates viral symptoms in TYLCV-infected tomato plants [71], as well as C4-triggered developmental abnormalities in transgenic *Arabidopsis* lines [63].

## Conclusions and outlook

Recent years have witnessed an unprecedented increase in publications featuring the geminiviral C4/AC4 protein, uncovering a plethora of functions and different properties of this positional homologue in different viruses; in all probability, however, we are just scratching at the surface of the diversity of this viral protein. Systematic comparative studies will be required to unveil the full breadth of the functional and mechanistic portfolio of the C4/AC4 proteins and their evolutionary underpinnings. Specifically, the annotated or predicted C4/AC4 homologues in members of the genera *Becurtovirus*, *Capulavirus*, *Grablovirus*, *Maldovirus*, *Mastrevirus*, *Mulcrilevirus*, *Opunvirus*, *Topilevirus*, and *Turncurtovirus* remain to be functionally characterized. Many members of these genera cause mild or symptomless infections, and it will be interesting to see if these viruses actually encode C4/AC4 proteins and, in case they do, to which extent these proteins are indeed functionally homologous to C4/AC4 proteins encoded by begomoviruses and curtoviruses.

Importantly, available examples in which the C4 coding sequence determines breakdown of resistance or independence from a satellite molecule underscore the importance of gaining insight into the C4/AC4 protein and its potential implications for disease control. Moreover, the identification of the virulence-promoting functions gained in C4/AC4 proteins may uncover processes required for a successful viral infection, which may be used as targets in strategies to engineer antiviral resistance, and the unraveling of their molecular bases may shed new light on plant cellular and molecular mechanisms. Last but not least, the exploitation of functional properties of geminiviral C4/AC4 proteins may entail high potential for biotechnological applications, e.g., in the targeted manipulation of plant signaling pathways. In all probability, research on these versatile and fascinating proteins will keep broadening our understanding of the intricate plant–geminivirus interactions, offering insight into geminiviral evolution, and, hopefully, propelling the rational design of antiviral strategies for years to come.

## Supporting information

S1 Table Scores and cutoffs for cTP, myr, and pal predictions in Table 1. cTP, chloroplast transit peptide; myr, myristoylation; pal, palmitoylation.
(XLSX)

S2 Table Features of the viral proteins selected for Fig 2. (XLSX)

S3 Table Nonsynonymous to synonymous substitution ratios (dN/dS) calculated for the 3 functional domains (cTP, chloroplast transit peptide; myr, myristoylation; pal, palmitoylation) of C4 proteins of 21 OW and NW begomoviruses. NW, New World, OW, Old World.
(DOCX)
