# Supplementary material for: Small but mighty: Functional landscape of the versatile geminivirus-encoded C4 protein
Source: PLoS Pathog. 2021 Oct 7;17(10):e1009915. doi: 10.1371/journal.ppat.1009915 (PMC8496806; doi:10.1371/journal.ppat.1009915)
Supplement: S3 Table — NW, New World, OW, Old World. (DOCX) [file ppat.1009915.s003.docx]

**S3 Table.** Non-synonymous to synonymous substitution ratios (dN/dS) calculated for the three functional domains (chloroplast transit peptide, cTP; myristoylation, myr; and palmitoylation, pal) of C4 proteins of 21 Old and New World begomoviruses.

| **Virus** | **Acronym** | **Group^a^** | **Number of sequences** | **AC4** | | | |
| --- | --- | --- | --- | --- | --- | --- | --- |
|  |  |  |  | **Full** | **cTP** | **myr** | **pal** |
| African cassava mosaic virus | ACMV | OW | 252 | **2.0018** | **1.8683** | nf^b^ | **1.0536** |
| Ageratum enation virus | AgEV | OW | 44 | 0.8989 | 0.7522 | **1.0832** | **1.2488** |
| Bhendi yellow vein India virus | BhYVIV | OW | 52 | **1.1987** | **1.0968** | 0.6770 | 0.8230 |
| Bhendi yellow vein mosaic virus | BhYVMV | OW | 75 | **1.4870** | 0.7828 | 0.8884 | **1.5723** |
| Cotton leaf curl Gezira virus | CLCuGV | OW | 81 | **1.4433** | **1.0215** | nf | **1.5420** |
| Cotton leaf curl Multan virus | CLCuMV | OW | 129 | **1.4449** | **1.2295** | 0.9379 | **1.1666** |
| East African cassava mosaic virus | EACMV | OW | 207 | **1.4322** | **1.1801** | 0.6008 | 0.9626 |
| Mungbean yellow mosaic India virus | MYMIV | OW | 85 | 0.5750 | nf | nf | **3.5136** |
| Squash leaf curl China virus | SLCCNV | OW | 38 | **1.4377** | 0.4159 | 0.1800 | nf |
| Sweet potato leaf curl virus | SPLCV | OW | 142 | **1.3603** | **1.2984** | 0.9944 | **1.2866** |
| Tomato leaf curl New Delhi virus | ToLCNDV | OW | 547 | 0.7223 | nf | 0.1897 | 0.3695 |
| Tomato leaf curl Taiwan virus | ToLCTV | OW | 54 | 0.9286 | **1.0111** | **1.0955** | **1.0395** |
| Tomato yellow leaf curl China virus | ToYLCCNV | OW | 39 | 0.9372 | nf | 0.3962 | 0.5632 |
| Tomato yellow leaf curl Thailand virus | TYLCTV | OW | 42 | 0.7963 | nf | 0.3377 | 0.4119 |
| Tomato yellow leaf curl virus | TYLCV | OW | 763 | **2.0298** | **3.3792** | 0.4575 | **2.9122** |
| Bean golden mosaic virus^c^ | BGMV | NW | 117 | **2.5300** | **2.3897** | **ssnf^d^** | nf |
| Blainvillea yellow spot virus^c^ | BlYSV | NW | 30 | 0.8575 | nf | 0.3111 | 0.7066 |
| Euphorbia yellow mosaic virus^c^ | EuYMV | NW | 50 | **1.1500** | 0.6190 | 0.1825 | 0.2094 |
| Macroptilium yellow spot virus^c^ | MaYSV | NW | 21 | **1.4500** | nf | 0.4010 | 0.7398 |
| Tomato leaf deformation virus | ToLDeV | NW | 72 | 0.5515 | 0.5221 | 0.3424 | 0.2899 |
| Tomato severe rugose virus | ToSRV | NW | 74 | **1.8367** | **3.5343** | **ssnf** | **ssnf** |

^a^OW, Old World; NW, New World

^b^nf, domain not found

^c^Values for the complete gene sequences from Xavier et al., 2020 [1]

^d^ssnf, synonymous substitutions not found, thus positive selection but dN/dS value cannot be calculated

**Reference:**

1. Xavier CAD, Godinho MT, Mar TB, Ferro CG, Sande OFL, Silva JC, et al. (2021) Evolutionary dynamics of bipartite begomoviruses revealed by complete genome analysis. Mol Ecol. 30: 3747-3767.
